# Some Clinical Cases

**Published:** 1889-06

**Authors:** R. M. Theobald

**Affiliations:** London


### SOME CLINICAL CASES.

R. M. Theobald, M. D., London.

(1) Miss R., aged thirty-three. December 28th, 1888.—Cracking in right ear when speaking ; deaf from a cold ; had discharge of pus and blood from ear some time ago ; low spirits ; stye in left-lower eyelid ; very much wind. *Phosphorus*$^{cm}$ (Fincke) one dose.

January 4th, 1889.—Better, but ear symptoms severe ; hissing like wind going into ear. Has had two styes, which have now gone.

January 21st.—Says it cured like magic. Quite well. *Indigo*$^{30}$, twice daily for two days.

(2) Mr. N. February 11th, 1889.—Rheumatic fever. Old rheumatic case, with heart disease and mitral regurgitation. Very passive, quiet disposition. No swelling or redness. Pain in moving, but afterward relieved by movement. *Rhus*$^{cm}$ (F. C.), two doses in twenty-four hours.

February 14th.—Much the same. *Sulphur*$^{cm}$ (Fincke), one dose. This cured; more progress in three days than in two months previously under allopathy.

(3) Mrs. H., aged twenty-nine. November 26th, 1887.—Thin, weak, emaciated. Leucorrhœa always profuse. Frequent diarrhœa. Menses delayed, scanty, painful. Appetite vanishes while eating. Heavy weight after food. Hysterical. Before any illness, sight becomes much clearer than usual—*i. e.*, things look bright. Giddy. Pain in left ovary. *Sulphur*$^{cm}$ (Fincke), two or three doses for one day.

December 10th.—Cervical glands swollen and painful. General symptoms same. *Lachesis*$^{cm}$ (F. C.), two or three doses, for one day.

December 17th.—Very much better and stronger. Glands less. Less diarrhœa or leucorrhœa. No pain in left ovary. Always heat at vertex.

December 24th.—Leucorrhœa has been very bad for one or two days. Other symptoms better. Repeat *Lachesis.*

December 31st.—Better. Repeat *Lachesis.*

January 11th, 1888.—Sick when beginning to eat. Diarrhœa three times daily. Cough with bits of tasteless mucus. Soon tired; very sleepy; low spirits; heavy headache; stomach feels swollen; pulsation in abdomen; hands burn and swell; feels cold. *Ignatia* for one day.

January 15th.—Giddy. Throbbing over right eye. Confused. More diarrhœa; sick and faint before and after it. *Lac caninum*$^{cm}$ (Fincke) for one day.

January 21st.—Better. Violent pain one night in left ovary. All symptoms better.

January 28th.—Not so well. Repeat *Lac can.*

February 3d.—Better. Diarrhœa still. Menses this week, bad, dirty color. Tongue white in morning. Very much flatulence. Throat sore at menses. Repeat *Lac caninum.*

February 10th.—Stronger; better in all respects. *Lac can.* one dose.

February 24th.—All symptoms better. Very little diarrhœa. Less leucorrhœa. No pain in left ovary. Pulsation in epigastrium. *Iodine* for one day.

March 9th.—Better. *Lac. caninum.* Gradually got well, with occasional doses of *Lac can.*

